# Chemopreventive Effect of Dietary Glutamineon Colitis-Associated Colorectal Cancer Is Associated with Modulation of the DEPTOR/mTOR Signaling Pathway

**DOI:** 10.3390/nu8050261

**Published:** 2016-05-02

**Authors:** Yun Tian, Keming Wang, Yingrui Fan, Yan Wang, Liqun Sun, Li Wang, Jirong Wang, Zhaoxia Wang, Juan Li, Ying Ye, Guozhong Ji

**Affiliations:** 1 Department of Oncology, the Second Affiliated Hospital of Nanjing Medical University, No. 121 Jiangjiayuan Road, Nanjing 210011, China; summer.cloud@live.cn (Y.T.); drkemingwang@sina.cn (K.W.); drfanyingrui@sohu.com (Y.F.); lixiang@kplink.cn (L.W.); rabbitwjr@126.com (J.W.); zhaoxiawang88@hotmail.com (Z.W.); ljsmz1229@163.com (J.L.); 2 Department of Pathology, the Second Affiliated Hospital of Nanjing Medical University, No. 121 Jiangjiayuan Road, Nanjing 210011, China; dryanwang@sina.com; 3 Department of Intensive Care Unit, the Second Affiliated Hospital of Nanjing Medical University, Nanjing 210011, China; drliqunsun@sina.com; 4 Emergency Center, Affiliated Hospital of Xuzhou Medical University, Xuzhou 221002, China; xzmcyy@163.com; 5 Institute of Digestive Endoscopy and Medical Center for Digestive Diseases, the Second Affiliated Hospital of Nanjing Medical University, No. 121 Jiangjiayuan Road, Nanjing 210011, China

**Keywords:** colitis, colorectal cancer, glutamine, DEPTOR, mTOR signaling

## Abstract

Glutamine plays a protective role in colitis and colitis-associated colorectal cancer (CAC); however, the protective mechanisms are largely unknown to date. DEP domain-containing mTOR-interacting protein (DEPTOR)/mammalian Target of Rapamycin (mTOR) signaling plays an important role in carcinogenesis. The present study investigated the potential molecular mechanisms for the protective effect of glutamine in a murine model of azoxymethane (AOM)/dextran sulfate sodium (DSS)-induced CAC. The effects of glutamine on DEPTOR/mTOR signaling and protein light chain 3 (LC3) were evaluated. Administration of glutamine was associated with attenuated development of CAC. Increased expression of DEPTOR and decreased expressions of factors of mTOR signaling, including phospho-mTOR, phospho-STAT3, phospho-Akt, and phospho-S6, were observed in AOM/DSS mice administered glutamine. Furthermore, oral glutamine was associated with increased LC3-II expression in AOM/DSS mice. The present study indicates that regulation of DEPTOR/mTOR signaling may be an important mechanism for glutamine in prevention against the development of CAC. In addition, the chemopreventive effect of dietary glutamine on CAC is, at least in part, associated with the induction of autophagy.

## 1. Introduction

Although significant efforts and intense investigations have been made to fight colorectal cancer (CRC) over the recent decades, CRC still ranks second in cancer-related deaths worldwide. More than 1.2 million patients are diagnosed with CRC, and greater than 600,000 deaths per year occur as a result of this disease. In addition to genetic heterogeneity, development of CRC through the adenoma—carcinoma sequence is affected by dietary habits and inflammation [1]. Chronic inflammation in the intestine ranks among the top three high-risk conditions for CRC, and inflammatory bowel disease (IBD) is an important risk factor for human CRC. Unfortunately, the mechanistic link between inflammation and tumorigenesis remains largely unknown [2].

Mammalian Target of Rapamycin (mTOR) resides in two distinct multi-protein complexes, referred to as mTOR complex 1 (mTORC1) and 2 (mTORC2) [3]. In general, mTORC1 controls mRNA translation, ribosome biogenesis, cell growth, and autophagy through regulating substrates, including S6K1 and 4E-BP1, whereas mTORC2 controls cell proliferation, cell survival, and the cytoskeleton through substrates, such as Akt, SGK1, and PGCα [3,4,5,6]. Deregulation of the mTOR signaling pathway has been demonstrated in a variety of human cancers, including CRC [7,8]. It was recently demonstrated that both mTOR complexes are directly inhibited by DEP domain-containing mTOR-interacting protein (DEPTOR), which binds and inhibits mTOR through a PDZ domain [9].

The beneficial effects of glutamine (GLN) have been described in many gastrointestinal disorders. In experimental animals with acetic acid-induced intestinal injury, pretreatment with oral GLN prevents mucosal injury and improves intestinal recovery [10,11]. *In vitro* experiments demonstrated that glutamine significantly reduced the permeability of the intestinal cell line [12]. In addition, mTOR activity and its downstream signaling is regulated by GLN [13,14,15]. Through the induction of autophagy via the regulation of the mTOR pathways, GLN contributes to cell survival during physiological stress [16]. Our previous data indicated a protective effect of GLN in the progression of a colitis-associated CRC (CAC) mouse model [17]. However, the mechanism remains unknown. Understanding the molecular mechanisms of GLN, which exhibits promising efficacy on both anticancer and anti-inflammatory activity, will aid in the management of CAC. Based on previous results, we aimed to explore the regulatory role of GLN on the DEPTOR-mTOR signaling pathway in an animal model of CAC.

## 2. Experimental Section

### 2.1. Animal Experiments

The azoxymethane (AOM)/dextran sulfate sodium (DSS) model of CAC was employed in this study. All experimental manipulations were performed in accordance with the institutional guidelines for the care and use of laboratory animals, and the study was approved by ethics committee of Nanjing Medical University. Our study included three groups: one control group, one DSS/AOM model of CAC group, and one GLN treated DSS/AOM model of CAC group (*n* = 10 for each group). The GLN-treated DSS/AOM model of CAC group was orally administered a GLN-enriched diet (3 g/100 kcal, Sigma–Aldrich, St. Louis, MO, USA) for one week at the beginning of the experiment and every other week thereafter, whereas mice in the other DSS/AOM group (model group) and control group received a control diet according to our experimental treatment protocol reported previously [17]. Mice receiving a GLN-enriched diet were orally administered 8.5 g GLN/kg/day, and the GLN-enriched diet was isonitrogenous and isocaloric (whey protein, 6 g; carbohydrate, 16 g; fat, 1.33 g; mineral, 1.91 g; cellulose, 0.76 g; vitamin, 0.38 g; glutamine, 3 g; glycine, 0 g).

### 2.2. Pathological Examination

Mice were sacrificed under anesthesia at day 90. The colon was excised from the ileocecal junction to the anal verge. Analysis of morphologic change, gross examination, and histological features was performed using the methods described in our previous study [17]. Colonic inflammation was evaluated and reported according to the histologic inflammatory score described by Cooper *et al.* [18].

### 2.3. Western Blot Analysis

Western blotting was performed as previously described [17]. Colon tissues were washed with Hank's balanced salt solution (HBSS). After washing, the tissue was homogenized in lysis buffer including a protease inhibitor cocktail table (Roche Diagnostics, Mannheim, Germany). An equal amount of protein (10–30 ng) was separated by 4%–20% SDS-PAGE, depending on the size of the proteins to be observed. Proteins were electrotransferred to PVDF membranes using a wet-transfer apparatus. Membranes were blocked with 5% nonfat milk in 1 × TBST for 1 h and then incubated with primary antibodies (DEPTOR, STAT3, and β-actin were purchased from Santa Cruz Biotechnology Inc., Delaware Ave Santa Cruz, CA, USA; mTOR, phosphor-mTOR, AKT, phosphor-AKT, S6, phosphor-S6, and light chain [LC]3 were purchased from Cell Signaling (Technology, Danvers, MA, USA) overnight at 4 °C. Membranes were washed thrice with 1 × TBST and incubated with horseradish peroxidase-conjugated species-specific antibodies for 1 h. Detection of protein expression was performed by incubating the membranes with ECL Plus (AMRESCO, AMRESCO LLC, 28600 Fountain Parkway Solon, OH, USA) and exposure to X-ray films.

### 2.4. Statistical Analyses

Continuous variables are presented as mean ± standard deviation (SD) tested using Student two-sample *t*-test for any two-sample comparisons. Comparisons between three groups were made by one-way ANOVA followed by Tukey’s or Dunnett’s *post hoc* multiple comparison tests. The Chi-squared test was used for comparison of the descriptive values. All *p*-values were two-sided. A *p-*value less than 0.05 was considered statistically significant.

## 3. Results

### 3.1. GLN Protects against CAC

Body weight was monitored once per week. Our data indicate that the body weight was reduced after the administration of DSS/AOM; however, mice in the GLN treated group exhibited increased body weight at weeks 2, 5, 6, 8, 9, 10, and 11 compared with the DSS/AOM group (*p* < 0.028). Oral GLN was associated with attenuated colorectal tumor development. Microscopic findings of inflammation in the colon and the incidence of colonic tumor are presented in Table 1. Oral administration of GLN was associated with a decreased inflammatory score in AOM/DSS treated mice (*p* = 0.010). Our data indicated that the administration of GLN significantly reduced the average number of adenomas (*p* = 0.028) compared with untreated AOM/DSS mice. In addition, treatment with GLN also significantly reduced the average number of adenocarcinomas compared with untreated AOM/DSS mice (*p* = 0.002). Furthermore, intake of GLN significantly decreased the average number of total tumors (*p* = 0.003) compared with untreated AOM/DSS mice.

### 3.2. GLN Promotes DEPTOR Expression

**Figure 1:**
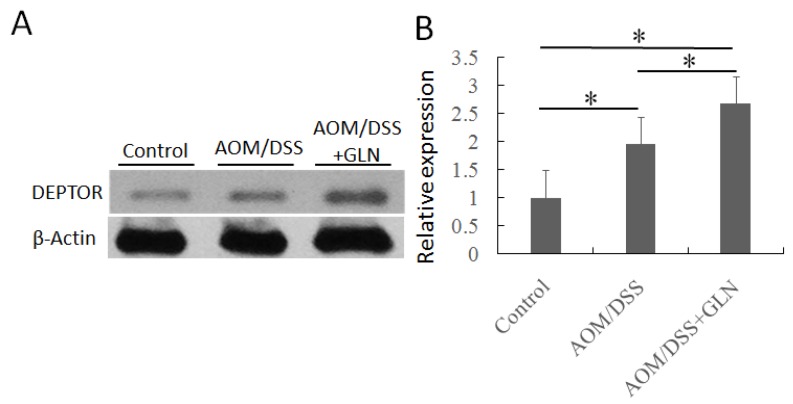
Changes in DEPTOR protein expression in the colon of mice. GLN significantly increased the protein expression in DSS/AOM mice with colitis-associated cancer (**A**). The expression of DEPTOR was statistically analyzed relative to β-actin expression by densitometry (**B**). Data are expressed as the means ± SD, * *p* < 0.05.

**Figure 2:**
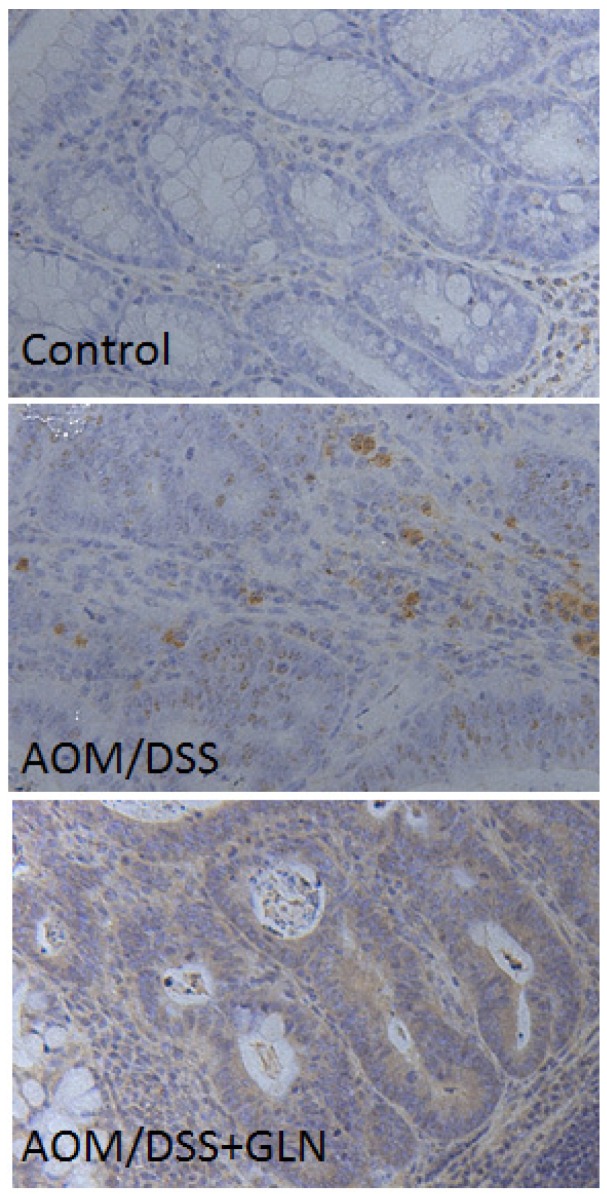
Effect of oral GLN on the expression of DEPTOR in DSS/AOM mice with colitis-associated cancer (×200). The expression of DEPTOR was found to be increased in DSS/AOM mice treated with GLN.

We first evaluated the impact of GLN administration on the expression of DEPTOR. Western blotting revealed that GLN-treated AOM/DSS mice exhibited increased DEPTOR expression compared with untreated AOM/DSS mice (Figure 1). Increased DEPTOR expression was also confirmed by the immunochemical study, which revealed increased DEPTOR expression in AOM/DSS mice treated with GLN (Figure 2).

### 3.3. GLN Regulates mTOR Signaling

**Figure 3:**
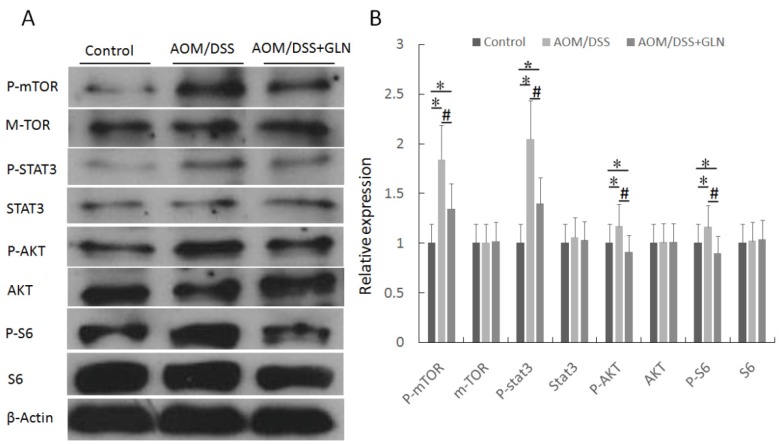
GLN suppresses m-TOR signaling pathway *in vivo*. Western blot analysis of P-mTOR, mTOR, P-STAT3, STAT3, P-AKT, AKT, P-S6, and S6 in AOM/DSS mice treated with or without GLN (**A**); and the expressions were statistically analyzed relative to β-actin expression by densitometry (**B**). Data are expressed as the means ± SD, * *p* < 0.05 *vs.* control group, # *p* < 0.05 *vs.* AOM/DSS group.

We next investigated the effects of oral GLN on the DEPTOR target protein mTOR. A significant decrease in mTOR phosphorylation was noted in AOM/DSS mice treated with GLN; however, the expression of the mTOR protein was comparable between AOM/DSS mice treated with and without GLN (Figure 3). We also examined whether GLN treatment was associated withSTAT3 inhibition. Our data revealed that oral GLN reduced STAT3 phosphorylation, whereas no significant difference was observed in STAT3 protein expression between GLN-treated and untreated AOM/DSS mice (Figure 3). In addition, P-AKT and P-S6 expression was significantly decreased in AOM/DSS mice receiving GLN (Figure 3).

### 3.4. GLN Induces Autophagy

**Figure 4:**
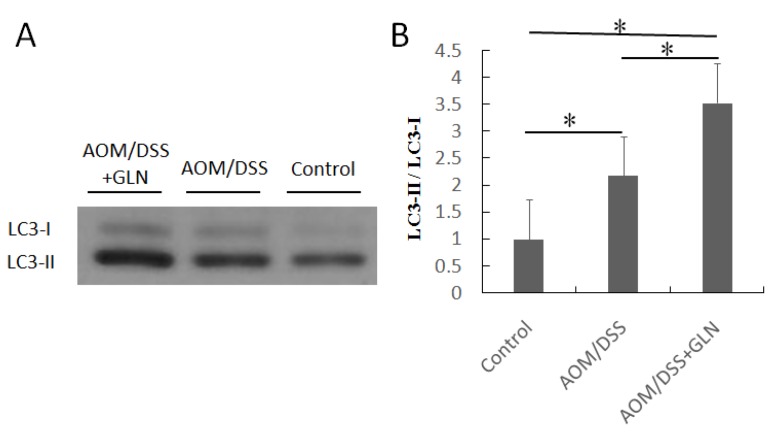
Effects of GLN on autophagy. Increased expression of LC3-II protein by GLN in AOM/DSS treated mice (**A**); The ratio of LC3-II/LC3-I proteins was statistically analyzed by densitometry (**B**). Data are presented as means ± SD. * Statistical differences with *p* < 0.05.

The mTOR pathway negatively regulates autophagy and, therefore, we assessed the effects of GLN on autophagy in the colons of AOM/DSS treated mice. Oral GLN induced LC3-II expression (Figure 4).

## 4. Discussion

The aim of this *in vivo* study was to explore the potential mechanisms of the chemopreventive effects of dietary GLN on CAC. Our study indicated that oral GLN was associated increased DEPTOR expression and inhibited the mTOR signaling pathway. In addition, enhanced autophagy, which is demonstrated by increased expression of LC3-II, was observed in AOM/DSS mice treated with GLN. Our data provide evidence that dietary GLN suppresses CAC development via mechanisms involving regulation of DEPTOR/mTOR signaling and autophagy induction.

Although multiple signaling pathways are involved in the pathogenesis of CRC, the molecular mechanisms of carcinogenesis remain poorly understood. Recently, the mTOR signaling pathway received increased attention because mTOR signaling is frequently hyper-activated in primary human CRC tumors [8]. The mTOR protein, an evolutionarily conserved serine/threonine kinase, is a member of the phosphoinositide-3-kinase (PI3K)-related family of kinases [5,6]. The mTOR is an important sensor of various metabolic and nutrient stresses, receiving and interpreting environmental inputs and transducing them to downstream signaling pathways [19]. In response to growth factor and nutrient signaling, mTOR plays a central role in the regulation of a variety of cellular processes, including cell growth, metabolism, autophagy and cell cycle progression [7,9,20]. Knockdown of mTOR leads to attenuated CRC tumor growth both *in vitro* and *in vivo*; thus, mTOR inhibitors could be promising anticancer therapeutic options in CRC treatment [8,21]. Additionally, mTOR and STAT3 pathways and their crosstalk are significantly upregulated in patients with IBD [22] and inhibition of mTOR ameliorated DSS-induced colitis [23].

DEPTOR has recently been identified as an endogenous mTOR inhibitor that could suppress both mTORC1 and mTORC2 activity *in vivo* [24]. DEPTOR is a critical determinant of mTOR pathway activity, and dysregulated DEPTOR might contribute to aberrant activation of mTOR in disease [25]. Through inhibition the mTORC1/PI3K signaling pathway, DEPTOR suppresses S6K1 and activates Akt [9]. In tumor development and progression, DEPTOR activity is associated with cell growth, apoptosis, autophagy, and the epithelial-mesenchymal transition [24]. Therefore, through mTOR’s inhibitory role, DEPTOR plays a pivotal role, at least in part, in the development and progression of human malignances. To date, the function of DEPTOR in tumorigenesis remains controversial. Targeting DEPTOR may be a novel strategy for anticancer therapy [24].

In our study, mice in the GLN group were orally administered a GLN-enriched diet that provides 8.5 g GLN/kg/day [26]. GLN administered to animals by oral gavage was associated with attenuated DSS-induced colitis [27]. In the present study, in the AOM/DSS-induced CAC mouse model, we observed a deregulation of DEPTOR-mTOR signaling and increased STAT3 activity. Interestingly, when the CAC model was induced, DEPTOR expression also increased. When a GLN-enriched diet was administrated, the colitis was statistically improved, and the tumor burden decreased. In addition, DEPTOR expression increased, and mTOR activity was partially restored. We hypothesized that GLN may be a partial inhibitor that reduced the rapidly degradation of DEPTOR. GLN also regulates the mTOR/p70 (S6) kinase pathway [28]. A negative correlation was noted between DEPTOR and mTOR expression, which was in consistent with the notion that DEPTOR is an endogenous mTOR inhibitor that could suppress mTOR activity. The mTOR signaling pathway is regulated by GLN [14,29]. Our study showed that GLN administration suppressed phosphorylation related to Akt/mTOR signaling, and S6 activity was also reduced by supplemental GLN. Activated STAT3, which could be trigged by mTOR, has been observed in CRC [30]. However, GLN treatment downregulated mTOR and inhibited STAT3. Mice treated with AOM/DSS exhibited increased expression of the DEPTOR protein compared with the control group, suggesting that DEPTOR is active in mice with AOM/DSS-induced CAC. Similarly, compared with mice in the control group, phospho-mTOR was significantly increased in mice treated with AOM/DSS. Interestingly, GLN-treated AOM/DSS mice exhibited reduced expression of phospho-mTOR and increased expression of DEPTOR compared with those treated with AOM/DSS alone. Increased DEPTOR expression in GLN-treated mice potentially results from a direct effect of GLN. Alternatively, these effects might be a consequence of decreased expression of phospho-mTOR given the positive feedback loop involving mTOR and DEPTOR [25]. If GLN has a direct active effect on DEPTOR expression, decreased activation of phospho-mTOR in the current study can be explained by the positive DEPTOR and mTOR feedback loop. Therefore, further *in vitro* studies are needed to confirm our results and to explore the potential direct effects of GLN on the DEPTOR/mTOR signaling in CAC.

mTOR is a well-characterized inhibitor of autophagy that is implicated in oncogenesis [14]. DEPTOR potentially induces autophagy by inhibiting mTOR activity [25]. In our study, the expression of LC3, which is a key molecule involved in autophagy, was increased. DEPTOR accumulation induced the expression of the lipidated species of the LC3 autophagy marker (referred to as LC3-II), suggesting that GLN-mediated anti-cancer effects in the DSS/AOM mouse model might be associated with enhanced autophagy. GLN involved in inhibition of the mTOR signaling pathway [14], thus potentially resulting in a subsequent increase in DEPTOR expression. We cannot exclude the possibility that glutamate is the decisive factor because apical glutamate effectively decreases permeability in colonic epithelial cell lines [31]. We have reported that GLN can suppress the activity of COX2, iNOS, and NF-κB in an DSS/AOM animal model. Thus, it is possible that the effect of GLN on the DEPTOR/mTOR pathway might result from the inhibition of the inflammatory response. A limitation of this study is the small number of animals in each group, which might reduce the power of our results; however, sensitive methods were applied. In addition, the 8.5 g GLN/kg/day dose was based on general conditions; therefore, it is possible that all mice did not receive this GLN dose. However, because GLN was orally administrated in this study, the precise dosage of GLN is difficult to determine.

## 5. Conclusions

In this study, disordered DEPTOR-mTOR signaling was noted in the CAC mouse model, whereas oral administration of GLN was involved in the regulation of this pathway, suggesting that the protect effects of GLN in CAC are at least partially mediated by modulation of DEPTOR-mTOR signaling. However, DEPTOR stability is largely governed by unknown mechanisms; thus, further studies focusing on this issue are needed.

## Figures and Tables

**Table 1:** Histological findings of colonic lesions in azoxymethane (AOM)/dextran sulfate sodium (DSS) mice and AOM/DSS mice receiving GLN.

	AOM/DSS	AOM/DSS + GLN	*p* Value
Colonic inflammation score	1.9 ± 0.7	1.0 ± 0.7	0.010
Adenoma, *n*/*N*, mean ± SD	8/10, 4.7 ± 3.3	6/10, 1.9 ± 1.7	0.028 ^a^
Adenocarcinoma, *n*/*N*, mean ± SD	10/10, 3.6 ± 1.7	5/10, 1.1 ± 1.3	0.002 ^a^
Total tumors, *n*/*N*, mean ± SD	10/10, 8.3 ± 4.1	7/10, 3.0 ± 2.6	0.003 ^a^

The “*n*” mean the number of mice with positive adenoma, adenocarcinoma, or tumors; the “*N*” means the total number of mice in each group; ^a^ *p*-value for mean ± SD between groups.

## Abbreviations

AOM, azoxymethane; CAC, colitis-associated colorectal cancer; CRC, colorectal cancer; DEPTOR, DEP domain-containing mTOR-interacting protein; DSS, dextran sulfate sodium; GLN, glutamine; IBD, inflammatory bowel disease; LC3, light chain 3; mTOR, mammalian Target of Rapamycin; PI3K, phosphoinositide-3-kinase.
